# HIV Incidence, Risk Factors, and Motivation for Biomedical Intervention among Gay, Bisexual Men, and Transgender Persons in Northern Thailand

**DOI:** 10.1371/journal.pone.0024295

**Published:** 2011-09-08

**Authors:** Suwat Chariyalertsak, Natthapol Kosachunhanan, Pongpun Saokhieo, Radchanok Songsupa, Antika Wongthanee, Chonlisa Chariyalertsak, Surasing Visarutratana, Chris Beyrer

**Affiliations:** 1 Research Institute for Health Sciences, Chiang Mai University, Chiang Mai, Thailand; 2 Ministry of Public Health, Chiang Mai Provincial Health Office, Chiang Mai, Thailand; 3 Department of Epidemiology, Johns Hopkins Bloomberg School of Public Health, Baltimore, Maryland, United States of America; The University of Hong Kong, Hong Kong

## Abstract

**Background:**

HIV prevalence among men who have sex with men (MSM) and transgender (TG) persons is high and increasing in Chiang Mai, northern Thailand.

**Objectives:**

To describe demographic, socioeconomic, sexual behavior and interest in future HIV prevention trials among gay and bisexual MSM and TG presenting for HIV testing (VCT) and pre-screening for the iPrEx pre-exposure chemoprophylaxis trail.

**Methods:**

In 2008–09, MSM/TG participants attending VCT were interviewed and tested for HIV and STI. Univariate and multivariate regression analyses were done to assess associations with HIV infection.

**Results:**

A total of 551 MSM clients (56.1% gay, 25.4% TG, and 18.5% bisexual (BS)) were enrolled. The mean age was 23.9 years. HIV prevalence among MSM overall was 12.9% (71/551); 16.5% among gay men, 9.3% among TG, and 6.9% among BS. Consistent use of condom was low, 33.3% in insertive anal sex and 31.9% in receptive anal sex. Interest in participation was high, 86.3% for PrEP, 69.7% for HIV vaccine trials, but 29.9% for circumcision. HIV was independently associated with being gay identified, aOR 2.8, p = 0.037 and with being aged 25–29, aOR 2.7, p = 0.027. Among repeat testers, HIV incidence was 8.2/100 PY, 95% CI, 3.7/100PY to 18.3/100PY.

**Conclusion:**

HIV risks and rates varied by self-reported sexual orientation and gender identity. HIV was associated with sexual practices, age, and being gay-identified. These are populations are in need of novel prevention strategies and willing to participate in prevention research.

## Introduction

HIV infection rates are high and rising among men who have sex with men (MSM) in Thailand [1], [2], [3]. Northern Thailand, and the northern capital, Chiang Mai City, has a severe epidemic among MSM and has both a traditional Transgender (TG) community and an emerging gay-identified community [4]. Chiang Mai City's *PIMAN* Center was a study site for the recent iPrEx trial of oral chemoprophylaxis among MSM and TG women [5]. iPrEx reported an overall 44% reduction in HIV incidence (95% CI, 15–63; P = 0.0005) among 2499 seronegative men and transgender women receiving oral daily Truvada vs placebo [5]. This landmark result has raised an array of new questions about the likely target populations for oral pre-exposure prophylaxis (PrEP,) and interest in communities at risk in this and other interventions, including next generation ARV-based regimens, HIV vaccines, and rectal microbicides [6].

The PIMAN Center provided HIV testing and counseling (VCT) and STI services to MSM in Chiang Mai as a gateway to contacting MSM and TG populations interested in participating in iPrEx and other HIV prevention trials. We report here on the population of MSM and TG who presented to the PIMAN Center seeking free HIV voluntary counseling and testing and/or STI services, during the iPrEx trial's recruitment period. The aim of this study was to assess the HIV prevalence among populations of MSM who self-identified as gay/homosexual, TG or bisexual; to describe their socio-demographics and sexual risk behavior patterns; and to assess willingness to participate in biomedical intervention trials for HIV prevention. Because clinical trial participants are highly selected populations, we report here on HIV rates and risks among all clients seeking VCT services to inform the potential rollout of PrEP in a developing country population of MSM and TG.

The “real world” interest in and uptake of PrEP and other ARV based approaches to HIV prevention is unknown [7]. Such interventions will likely require targeting to populations at substantial risk, and during key periods in the life cycle. HIV risks are fluid, complex, and culturally bound, requiring careful assessment in differing cultural and risk environments. Northern Thailand has long had a distinct cultural identity, and a well-described tradition of tolerance for transgender persons known in Thai as *Katoey* [4]. Katoey are biological males who typically adopt female gender identities in childhood or early adolescence, speak the female dialect of Thai, and adopt women's names, dress, and identities. In the modern era, Thai *Katoey* have commonly used hormone therapy, surgical interventions, and other tools of modern medicine to assist in their female gender identity.

Bisexual behavior is also relatively common among Thai men, and being bisexual is now a recognized sexual orientation (*Seua Bi*) [8]. The emergence of an “out” modern gay identity is relatively new in Thai culture, but is now well established in urban areas, including Chiang Mai, which has a number of gay bars, clubs, and saunas, as well as community-based LGBT and gay men's organizations and groups [9]. The PIMAN clinic has aimed to provide a safe and welcoming environment for gay, bisexual, TG and other MSM, and is known as a gay/MSM-friendly space for health services.

## Methods

The *PIMAN* Center (**P**revention of **I**nfection in **Man**) is the only MSM-friendly service clinic providing free VCT and STI services for MSM and TG in Chiang Mai. It is located in the downtown area, popular among the MSM population in Chiang Mai. The Clinic is near Chiang Mai University, the largest in the region, and other public and private universities and colleges, in an area with more than 50,000 students. The clinic started operation in April, 2008. The clinic was a site for screening and recruitment of MSM and TG volunteers into the iPrEX trail and began enrolling in the trial in February 2009. An open label assessment among trial participants in iPrEx is ongoing at this writing. The *PIMAN* VCT service clinic was run as a pre-screening site supported by the Research Institute for Health Sciences (RIHES), Chiang Mai University, to seek potential volunteers who might be interested in participation in HIV prevention trials, including a proposed HIV vaccine trial in 2013–14.

At the clinic, all clients needed to be older than 18 years of age and were asked to provide written informed consent prior to receiving pretest counseling. They underwent HIV antibody testing if they wished to proceed. Clients \]who presented with any STI related signs or symptoms were offered examination by on-site clinicians, and laboratory investigations were performed for diagnosis syphilis by rapid plasma regain (RPR; Macro-Vac TM RPR Card Tests, Becton Dickinson) and confirmed by the treponema pallidum particle agglutination test (TPPA ; SERODIA- TPPA, Fujirebio Inc, Japan) and diagnosis of GC by performing gram stain from urethral discharge.

All clients were invited to come back within one week for post test counseling and then informed of their HIV test results. Those who found to be HIV antibody positive were given information on options for care and treatment, and on where to obtain clinical assessment and access to antiretroviral treatment (ART). (Thailand has had a national policy of universal access to ARVs since 2005 and Chiang Mai University Hospital is a leading provider of ARV care through this program). They were also informed about other ART clinical trials underway at RIHES and for which they might be candidates (these included HPTN052, HPTN063, and several ACTG trials). Those with negative HIV results received individualized risk reduction counseling for HIV prevention and were informed about the iPrEx trial; those interested were referred to the iPrEx recruiters on site. Clients who reported actively engaging in HIV risk behaviors were also invited to come back for HIV testing every 3 to 6 months.

VCT clients who agreed to participation in this study were asked to provide information on a short demographic and risk factor assessment. Information obtained included socio demographics, sexual risk behaviors and condom use, history of taking drugs and alcohol consumption, history of STIs, VCT uptake, and willingness to participate in the biomedical intervention trials for HIV prevention including OP trials, vaccine trials and trials of male circumcision. This study was reviewed and approved by the Human Ethics Committee of the Research Institute for Health Sciences, Chiang Mai University.

### Laboratory HIV testing

Serum was tested for HIV antibodies using Determine HIV-1/2 (Abbott). Positive sera were confirmed by ELISA. Discrepant results were tested with a tie-breaker, this third test was done with Gelatin participate agglutination (GPA).

### Data analysis

The data were analyzed using the software Stata/IC for Windows Version 10.0 (StataCorp LP, Texus, USA)). Data were presented as means, median or percentages in tables. Multivariate analysis using logistic regression was performed to assess factors predictive of HIV infection.

## Results

From April 2008 to December 2009, 551 MSM and TG clients presented for HIV VCT at the *PIMAN* Clinic.,. We present here the data from the initial HIV screening visit on all 551 clients who consented to HIV testing. Among persons screened, 78 were subsequently successfully enrolled into the iPrEx trial. There were 630 VCT services delivered in this period, with 11.4% of MSM clients having had more than 1 VCT episode at the clinic. The data from each client's first VCT service were used for the risk factor analysis.

A subset of men, 81 in all, returned for a second VCT episode after an initial negative HIV test. Of these, 6 seroconverted between their first and a subsequent VCT episode, allowing for an estimate of incident infections. Using a midpoint assumption for seroconversion and a 100 person/years at risk approach, the estimated incidence among repeat testers was 8.2/100PY, 95% CI 3.7/100PY, 18.3/100PY. It is likely that repeat testers represent a relatively high risk subset of MSM, but the number of incidence cases was too small to assess risk differences.

### Demographics

Table 1 shows demographic variables, history of HIV testing, and HIV prevalence rates by self reported sexual orientation and gender identity among 551 MSM and TG. Clients of VCT fell generally into three categories: self-identified gay men 56.1%; transgenders, TG, (*Katoey*,) 25.4% (of whom only two reported being post-operative TG women); and bisexual men (BM), 18.5%. The mean age was 24.5 in Gay and BM and slightly younger, 22.4 years, in TG. Overall, 70.2% were aged 24 or below, and only 11.3% of all men were aged 30 or older. Some 81.3% identified as single, 10.7% as married men who had sex with men only, and 6.7% as married in men who have sex with men and women. Gay and TG clients were better educated than the BM (60.8% in Gay and 50.7% in TG had studied at the bachelor degree or higher, while only 24.5% of BM had the same educational level.) Student status was more common among Gay (41.1%) and TG (49.3%) men than among BM, at 16.7%. Being a non-Thai citizen was most common among BM (25.5%).

**Table 1:** Demographics, history of HIV testing and HIV prevalence among Gay and Bisexual MSM, and Transgendered persons in Chiang Mai Thailand, 2010.

Characteristics	Sexual orientation, gender identity						Total	
	Gay		TG		Bisexual			
	N	*%*	N	*%*	N	*%*	N	*%*
Total cases	309	*56.1*	140	*25.4*	102	*18.5*	551	*100.0*
**Age (yrs.)** *χ^2^(6) = 25.20, p = 0.000*								
*Mean/Median (Range)*	*24.5/23(18–53.5)*		*22.4/21(18.1–47.9)*		*24.5/23.2(18–55.7)*		*24/22.5(18–55.7)*	
18–19	**61**	*19.7*	**52**	*37.1*	**24**	*23.5*	**137**	*24.9*
20–24	**142**	*46.0*	**65**	*46.4*	**43**	*42.2*	**250**	*45.4*
25–29	**63**	*20.4*	**14**	*10.0*	**25**	*24.5*	**102**	*18.5*
30 or more	**43**	*13.9*	**9**	*6.4*	**10**	*9.8*	**62**	*11.3*
**Marital Status** *χ^2^(6) = 71.89, p = 0.000*								
Single	**256**	*83.1*	**115**	*82.1*	**76**	*74.5*	**447**	*81.3*
Married (sex with male only)	**34**	*11.0*	**25**	*17.9*	**0**	*0.0*	**59**	*10.7*
Married (sex with both male and female)	**14**	*4.5*	**0**	*0.0*	**23**	*22.5*	**37**	*6.7*
Divorced/Widowed	**4**	*1.3*	**0**	*0.0*	**3**	*2.9*	**7**	*1.3*
**Level of education** *χ^2^(4) = 61.63, p = 0.000*								
Secondary or less	**83**	*26.9*	**46**	*32.9*	**70**	*68.6*	**199**	*36.1*
Vocational	**38**	*12.3*	**23**	*16.4*	**7**	*6.9*	**68**	*12.3*
Bachelor or more	**188**	*60.8*	**71**	*50.7*	**25**	*24.5*	**284**	*51.5*
**Occupation** *χ^2^(6) = 38.44, p = 0.000*								
Student	**127**	*41.1*	**69**	*49.3*	**17**	*16.7*	**213**	*38.7*
Employ	**160**	*51.8*	**51**	*36.4*	**72**	*70.6*	**283**	*51.4*
Sex worker	**7**	*2.3*	**8**	*5.7*	**7**	*6.9*	**22**	*4.0*
Jobless	**15**	*4.9*	**12**	*8.6*	**6**	*5.9*	**33**	*6.0*
**Ethnicity** *χ^2^(2) = 58.47, p = 0.000*								
Thai	**292**	*94.5*	**140**	*100.0*	**76**	*74.5*	**508**	*92.2*
Non Thai (Ethnic minority)	**17**	*5.5*	**0**	*0.0*	**26**	*25.5*	**43**	*7.8*
**HIV testing History** *χ^2^(2) = 11.57, p = 0.003*								
No	**123**	*39.8*	**79**	*56.4*	**41**	*40.2*	**243**	*44.1*
Yes	**186**	*60.2*	**61**	*43.6*	**61**	*59.8*	**308**	*55.9*
**If yes :**								
**Last HIV test/VCT**								
Less than 1 yr	**117**	*63.9*	**38**	*62.3*	**42**	*68.9*	**197**	*64.6*
Longer than 1 year	**66**	*36.1*	**23**	*37.7*	**19**	*31.1*	**108**	*35.4*
**Reason to have HIV test in the past**								
Self risk	**99**	*53.2*	**32**	*52.5*	**36**	*59.0*	**167**	*54.2*
Employment	**35**	*18.8*	**10**	*16.4*	**13**	*21.3*	**58**	*18.8*
Blood donation	**52**	*28.0*	**19**	*31.1*	**12**	*19.7*	**83**	*26.9*
**HIV test result at the first visit** *χ^2^(2) = 8.52, p = 0.014*								
Negative	**258**	*83.5*	**127**	*90.7*	**95**	*93.1*	**480**	*87.1*
Positive	**51**	*16.5*	**13**	*9.3*	**7**	*6.9*	**71**	*12.9*
**Post test counseling service** *χ^2^(2) = 3.69, p = 0.158, NS*								
Did not return for test results	**25**	*8.1*	**7**	*5.0*	**12**	*11.8*	**44**	*8.0*
Returned for test results	**284**	*91.9*	**133**	*95.0*	**90**	*88.2*	**507**	*92.0*

### Sexual risk behaviors, male circumcision, and condom use

Table 2 shows the prevalence of sexual behaviors and substance use risks. Alcohol was the most commonly used substance, followed my methamphetamine use, at some 11.1% of all men. Substance use and alcohol did not appear to differ substantially by sexual orientation or gender identity. Self-reported STI were uncommon, except among BM, a significant proportion of whom also reported exchange sex (59.8%), suggesting that some may be sex workers with higher risks than Gay or TG persons.

**Table 2:** Behavioral factors, substance use, STI and sexual risks among Gay, Transgender, and Bisexual MSM in Chiang Mai, 2010.

Factors	Sexual orientation gender identity						Total	
	Gay		TG		Bisexual			
	N	*%*	N	*%*	N	*%*	N	*%*
Total cases	309	*56.1*	140	*25.4*	102	*18.5*	551	*100.0*
**Alcohol consumption**								
Never/Ever but now not active drinking	**45**	*15.0*	**20**	*14.7*	**7**	*7.1*	**72**	*13.5*
Active drinking	**255**	*85.0*	**116**	*85.3*	**92**	*92.9*	**463**	*86.5*
*χ^2^(2) = 4.26, p = 0.12, NS*								
<1 time/week or occasional	**119**	*47.0*	**61**	*53.0*	**33**	*36.3*	**213**	*46.4*
1–3 times/week	**102**	*40.3*	**31**	*27.0*	**32**	*35.2*	**165**	*35.9*
4–6 times/week	**13**	*5.1*	**7**	*6.1*	**6**	*6.6*	**26**	*5.7*
≥7 times/week	**19**	*7.5*	**16**	*13.9*	**20**	*22.0*	**55**	*12.0*
*χ^2^(6) = 19.54, p = 0.003*								
**Using substance abuse (Ever)**								
Never	**271**	*87.7*	**124**	*88.6*	**68**	*66.7*	**463**	*84.0*
Methamphetamine	**25**	*8.1*	**14**	*10.0*	**22**	*21.6*	**61**	*11.1*
Marihuana	**9**	*2.9*	**1**	*0.7*	**6**	*5.9*	**16**	*2.9*
Heroin	**1**	*0.3*	**1**	*0.7*	**0**	*0.0*	**2**	*0.4*
Metamphetamine+Marihuana	**2**	*0.6*	**0**	*0.0*	**6**	*5.9*	**8**	*1.5*
Metamphetamine+Heroin	**1**	*0.3*	**0**	*0.0*	**0**	*0.0*	**1**	*0.2*
*χ^2^(10) = 41.55, p = 0.000*								
**Self reported STD**								
Never in life time	**265**	*85.8*	**134**	*97.1*	**75**	*73.5*	**474**	*86.3*
Yes, more than 6 months	**33**	*10.7*	**3**	*2.2*	**14**	*13.7*	**50**	*9.1*
Yes, last 6 months	**11**	*3.6*	**1**	*0.7*	**13**	*12.7*	**25**	*4.6*
*χ^2^(4) = 34.46, p = 0.000*								
**Male circumcision**								
No	**280**	*90.9*	**129**	*93.5*	**97**	*95.1*	**506**	*92.3*
Yes	**28**	*9.1*	**9**	*6.5*	**5**	*4.9*	**42**	*7.7*
*χ^2^(2) = 2.24, p = 0.33, NS*								
**Sex exchange for money & goods**								
No	**244**	*79.0*	**113**	*80.7*	**41**	*40.2*	**398**	*72.2*
Yes	**65**	*21.0*	**27**	*19.3*	**61**	*59.8*	**153**	*27.8*
*χ^2^(2) = 64.20, p = 0.000*								

Table 3 show sexual partner numbers and type, and sexual practices. As expected, sex with women was most commonly reported by bisexually identified men, of whom 71.6% reported this behavior. Sex with women was uncommon among Gay men (8.1%) and very rare among TG (1.4%). Most participants (90%) reported sex with male sex partners, either regular or casual sex partners, in the previous 6 months. Only a small proportion, 1.6%, had more than 5 regular male partners in the previous 6 months. For casual male sex partners, 25% had between 2–4, 16.3% had between 5–10, and 12.9% reported more than 10 in the past 6 months, which was highest in BM.

**Table 3:** Sexual relationships and condom use among MSM and TG, Chiang Mai 2010.

Factors	Sexual orientation gender identity						Total	
	Gay		TG		Bisexual			
	N	*%*	N	*%*	N	*%*	N	*%*
Total cases	309	*56.1*	140	*25.4*	102	*18.5*	551	*100.0*
**Last 6 months: Number of female sex partner(s)** *χ^2^(8) = 259.64, p = 0.000*								
*Mean/Median (Range)*	*0.11/0(0–3)*		*0.01/0(0–1)*		*2.21/1(0–30)*		*0.48/0(0–30)*	
None	**284**	*91.9*	**138**	*98.6*	**29**	*28.4*	**451**	*81.9*
1	**18**	*5.8*	**2**	*1.4*	**27**	*26.5*	**47**	*8.5*
2–4	**7**	*2.3*	**0**	*0.0*	**35**	*34.3*	**42**	*7.6*
5–10	**0**	*0.0*	**0**	*0.0*	**9**	*8.8*	**9**	*1.6*
>10	**0**	*0.0*	**0**	*0.0*	**2**	*2.0*	**2**	*0.4*
**Last 6 months: Number of regular male sex partner(s)** *χ^2^(6) = 26.99, p = 0.000*								
*Mean/Median (Range)*	*0.83/1(0–10)*		*0.77/1(0–10)*		*0.48/0(0–6)*		*0.75/1(0–10)*	
None	**131**	*42.4*	**62**	*44.3*	**72**	*70.6*	**265**	*48.1*
Yes	**178**	*57.6*	**78**	*55.7*	**30**	*29.4*	**286**	*51.9*
1	**139**	*45.0*	**64**	*45.7*	**21**	*20.6*	**224**	*40.7*
2–4	**33**	*10.7*	**12**	*8.6*	**8**	*7.8*	**53**	*9.6*
5–10	**6**	*1.9*	**2**	*1.4*	**1**	*1.0*	**9**	*1.6*
**Anal sex with regular male Sex partner(s)** *χ^2^(4) = 135.53, p = 0.000*								
Having anal sex (AS) with regular male sex partner(s)	**172**	*96.6*	**78**	*100.0*	**29**	*96.7*	**279**	*97.6*
Insertive only	**51**	*29.7*	**1**	*1.3*	**23**	*79.3*	**75**	*26.9*
Receptive only	**58**	*33.7*	**76**	*97.4*	**1**	*3.4*	**135**	*48.4*
Both	**63**	*36.6*	**1**	*1.3*	**5**	*17.2*	**69**	*24.7*
Other types of sexual act but no AS	**6**	2.3	**0**	0.0	**1**	3.3	**7**	2.4
**Last 6 months: unprotected anal intercourse with regular male sex partner(s)** * *χ^2^(2) = 4.81, p = 0.09*								
No (Used condom all the times)	**44**	*25.6*	**20**	*25.6*	**13**	*44.8*	**77**	*27.6*
Yes (Not used all the times)	**128**	*74.4*	**58**	*74.4*	**16**	*55.2*	**202**	*72.4*
**Last 6 months: Number of casual male sex partner(s)** *χ^2^(8) = 16.36, p = 0.038*								
*Mean/Median (Range)*	*5.28/2(0–100)*		*20.22/1.5(0–899)*		*7.67/2(0–103)*		*9.52/2(0–899)*	
None	**104**	*33.7*	**43**	*30.7*	**26**	*25.5*	**173**	*31.4*
Yes	**205**	*66.3*	**97**	*69.3*	**76**	*74.5*	**378**	*68.6*
1	**35**	*11.3*	**27**	*19.3*	**17**	*16.7*	**79**	*14.3*
2–4	**81**	*26.2*	**36**	*25.7*	**21**	*20.6*	**138**	*25.0*
5–10	**57**	*18.4*	**14**	*10.0*	**19**	*18.6*	**90**	*16.3*
>10	**32**	*10.4*	**20**	*14.3*	**19**	*18.6*	**71**	*12.9*
**Anal sex with casual male Sex partner(s)** *χ^2^(4) = 234.63, p = 0.000*	**197**	*96.1*	**92**	*94.8*	**71**	*94.7*	**360**	*95.5*
Insertive only	**54**	*27.4*	**1**	*1.1*	**58**	*81.7*	**113**	*31.4*
Receptive only	**46**	*23.4*	**84**	*91.3*	**1**	*1.4*	**131**	*36.4*
Both	**97**	*49.2*	**7**	*7.6*	**12**	*16.9*	**116**	*32.2*
Other types of sexual act	**8**	*3.9*	**5**	*5.2*	**4**	*5.3*	**17**	*4.5*
**Last 6 months: unprotected anal intercourse (UAI) with casual male sex partner(s)** * *χ^2^(2) = 18.29, p = 0.000*								
No (Used condom all the times)	**103**	*52.8*	**41**	*44.6*	**54**	*77.1*	**198**	*55.5*
Yes (Not used all the times)	**92**	*47.2*	**51**	*55.4*	**16**	*22.9*	**159**	*44.5*
**Last 6 months : Anal sex with regular or casual male Sex partner(s)** *χ^2^(4) = 289.42, p = 0.000*	**276**	*96.8*	**118**	*95.9*	**83**	*95.4*	**477**	*96.4*
Insertive only	**72**	*26.1*	**1**	*0.8*	**66**	*79.5*	**139**	*29.1*
Receptive only	**69**	*25.0*	**109**	*92.4*	**2**	*2.4*	**180**	*37.7*
Both	**135**	*48.9*	**8**	*6.8*	**15**	*18.1*	**158**	*33.1*
Other types of sexual acts	**9**	*3.2*	**5**	*4.1*	**4**	*4.6*	**18**	*3.6*
**Last 6 months: unprotected anal intercourse with regular or casual male sex partner(s)** * *χ^2^(2) = 30.87, p = 0.000*								
No (Used condom all the times)	**99**	*35.9*	**33**	*28.0*	**54**	*65.1*	**186**	*39.0*
Yes (Not used all the times)	**177**	*64.1*	**85**	*72.0*	**29**	*34.9*	**291**	*61.0*

*regular or casual male sex partners that are insertive, receptive, or both.

Regular or steady partners were common and were reported by 57.6% of Gay men, 55.7% of TG and 29.4% in BM. Among these partnered men, 97.6% reported having had anal sex. Strictly insertive anal sex positioning was reported by most BM, 79.3%, while strictly receptive anal sex was the predominant behavior reported by TG, 97.4%, p<.0001. Gay men, in contrast to both other groups, were much more likely to report engaging in both insertive and receptive anal sex, p<. 0001. Only 27.6% of those who had anal sex with regular male partners reported use of condoms all the time.

Having sex exchange for money or goods in the last 6 months was reported by 27.8% of the men overall, but was much more common for BM, at 59.8%, p<.0001. suggesting many of these may be sex workers. Paying for sex in the last 6 months was uncommon, and reported by only 7.6% of participants.

### History of VCT uptake, STI and HIV prevalence

A history of ever having had an HIV test or VCT was reported by 55.9% of our clients, and was more common among Gay and BM (60.2% and 59.8% respectively) than among TGs, at 43.6%. Among those who ever had an HIV test, 64.6% had been tested in the past year. The most common reasons for testing were self-perceived high risk behavior 54.2%, having donated blood 26.9%, and been required by their business or health insurance, 18.8%.

Self reported lifetime history of STI diagnoses were reported by 13.7%, but only 4.6% reported an STI in the last 6 months before the visit. At the time of clinic visits, 40 (7.3%) had any signs or symptoms related to STI, and 22 agreed to see the clinicians. Four cases of syphilis and 4 of GC were confirmed with laboratory diagnostics (one participant had both syphilis and GC).

All 551 MSM clients underwent for HIV testing after pretest counseling. The overall HIV prevalence was12.9%; highest in Gay men and lowest in BM (16.5% in Gay, 9.3% in TG and 6.9% in BM). Encouragingly, 507 (92.0%) returned for post test counseling and return rates were high among all groups; 91.9% of Gay men, 95.0% of TG and 88.2% of BM.

### Willingness to participate in biomedical HIV prevention trials

An assessment of willingness to participate in biomedical HIV prevention trials was included 3 months after VCT services began, and data are available for 468 clients who presented for VCT after this module was added to the intake assessment. Among these 468 clients, 86.3% reported interest in oral PreP trials, 69.7% in HIV vaccine trials and only 29.9% in male circumcision trials. The data showed that Gay and TG men were more interested in participation in trials compared with BM (data not shown), but no significant associations with risk or other variables were associated with interest in participation.

### Risk Factors for HIV Infection

We conducted univariate and multivariate logistic regression analyses to assess independent risks for prevalent HIV infection among these men.(Table 4) Gay identified men were significantly more likely to have HIV infection than BM or TGs, aOR 2.8, p = 0.037. Men aged 25–29 were also at significantly higher risk than younger or older men, aOR 2.7, p = 0.027. Marital status, educational level, occupation and ethnicity were not associated with HIV infection risks.(Data not shown).

**Table 4:** Univariate and multiple logistic regression analysis of risk factors for HIV infection among MSM in Chiang Mai, Thailand, 2010.

Characteristics	Total cases	Seropositive		OR	95% CI	*P-value*	*Final Model*		
		N	%				AOR	95%CI	P-value
**Gender identity**									
Gay	309	51	**16.5**	**2.68**	**1.16–7.24**	**0.015**	**2.80**	**1.06–7.34**	**0.037**
TG	140	13	**9.3**	1.39	0.49–4.27	*0.50* *	1.09	0.33–3.57	0.89*
Bisexual	102	7	**6.9**	1					
**Age (yrs)**									
18–19	137	10	7.3	1					
20–24	250	32	12.8	1.86	0.86–4.39	**0.096**	1.85	0.85–4.05	0.122
25–29	102	21	20.6	**3.29**	**1.39–8.21**	**0.003**	**2.69**	**1.12–6.49**	**0.027**
≥30	62	8	12.9	1.88	0.61–5.61	*0.20* *	1.58	0.54–4.60	0.41*
**Marital Status**									
Single	447	61	13.6	2.77	0.68–24.29	*0.15* *			
Married (sex with male only)	59	7	11.9	2.36	0.41–24.34	*0.29* *			
Married (sex with both male and female)	37	2	5.4	1					
Divorced/Widowed	7	0	0.0						
**Post test counseling service**									
Not returning for test results	44	10	22.7	**2.15**	**0.90–4.73**	**0.042**			
Returning for test results	507	61	12.0	1					
**Using substance abuse (Ever)**									
Never	463	56	12.1	1					
Metamphetamine	61	12	19.7	**1.78**	**0.81–3.65**	***0.098***			
Marihuana	16	1	6.3	0.48	0.01–3.27	*0.48* *			
Heroin¥	2	1	50.0	**7.27**	**0.09–571.8**	***0.10***			
Metamphetamine+Marihuana	8	0	0.0						
Metamphetamine+Heroin¥	1	1	100.0						
**Sex exchange for money & goods**									
No	398	43	10.8	1					
Yes	153	28	18.3	**1.85**	**1.06–3.19**	***0.019***	**1.90**	**0.95–3.77**	**0.068**
**Anal sex with regular male sex partner(s)**	279	32	11.5						
Insertive	75	3	4.0	1					
Receptive	135	21	15.6	**4.42**	***1.25–23.84***	***0.012***			
Both	69	8	11.6	**3.15**	***0.71–19.07***	***0.087***			
**In last 6 months: Number of casual male sex partner(s)**									
None	173	15	8.7	1					
Yes	378	56	14.8						
1	79	6	7.6	*0.87*	*0.26–2.48*	*0.77* *	*1.16*	*0.35–3.85*	*0.81* *
2–4	138	17	12.3	*1.48*	*0.66–3.32*	*0.29* *	*1.67*	*0.62–4.49*	*0.31,* *
5–10	90	15	16.7	***2.11***	***0.90–4.88***	***0.053***	*2.33*	*0.81–6.75*	*0.12* *
>10	71	18	25.4	***3.58***	***1.57–8.17***	***0.0005***	***4.09***	***1.31–12.73***	***0.015***
**Anal sex with casual male sex partner(s)**	360	54							
Insertive	113	9	8.0	*1*					
Receptive	131	18	13.7	*1.84*	*0.74–4.86*	*0.15,* *			
Both	116	27	23.3	***3.51***	***1.49–8.89***	***0.002***			
Other types of sexual act	17	2	11.8						

*Not significant;

¥ Heroin (injecting and inhaled).

Men who reported past HIV testing were not more or less likely to have prevalent HIV infection, but men who reported testing and not returning for HIV test results were nearly twice as likely to be HIV infected on presentation to the clinic and this association reached borderline significance, p = 0.042.

Alcohol use was not associated with HIV infection, and the great majority of men across groups reported some alcohol use, but use of methamphetamine and heroin, rare overall, were much more common among HIV positive men, with methamphetamine reaching borderline significance.(Table 4)

STI self-reports were not associated with HIV infection. Being circumcised, reported by 42 of 548 men, 7.7%, was associated with HIV infection, and was statistically significant, though the numbers of HIV + circumcised men were small (N = 11) and the association likely spurious.

Sex with women was not associated with HIV infection among these men, nor were numbers of regular male partners in the previous 6 months. Reporting only receptive anal sex or both receptive and insertive sex with regular male partners were both independent risks for HIV infection. Men reporting exclusively receptive behaviors with regular partners were some 4 fold more likely to have HIV, p = 0.012.

Having had casual male sex partners was not associated with HIV risks, except for those men with more than 10 casual sex partners in the past 6 months, aOR 4.1, p = 0.015. As with regular partners, men reporting both receptive and insertive anal sex with casual partners were more likely to have HIV.

## Discussion

We have identified a young, well educated, but markedly at-risk population of MSM and TG seeking HIV testing in Northern Thailand. Despite being well informed about HIV, and having actively sought HIV testing, these young men continue to engage in sufficient HIV risk behaviors and to have substantial HIV infection rates. Self-identified Gay men were significantly more likely to have HIV infection than Bisexual men, and Gay men were the majority of our sample, a marked change in Thai culture from earlier studies where “out” gay identities were uncommon [10]. The HIV prevalence among these young gay men was higher than for any other group, likely reflecting the high rates of receptive anal intercourse and relatively low condom use rates reported by these men. The relatively large (N = 140) Transgender population had intermediate, but still high, HIV infection rates. There was a striking divergence in sexual practices reported by these men by sexual orientation and gender identity with 97.4% of TG individuals reporting practicing exclusively receptive anal intercourse, while 79.3% of Bisexual men reported exclusively insertive anal sex practices. Thai Gay men, in contrast, commonly reported both insertive and receptive anal sex practices, and with both stable and casual partners. These differences in sex practices by orientation and gender identity have important preventive implications, as TG and Gay identified men are clearly targets for interventions aimed at increasing condom use, and for risk reduction strategies including oral pre-exposure prophylaxis and topical (rectal) microbicide research, and HIV vaccine research.

A relatively small subset of MSM had more than one VCT episode, allowing for an estimate of HIV incidence, which was quite high, at some 8.2/100 PY. This incidence estimate must be interpreted with caution. Only 11% of MSM were repeat testers, so this was a subset of men who may have been at greater risk for HIV, or who may have returned for VCT due to known or suspected risk exposures or sero-conversion symptoms. Prospective data on a less selected population of MSM and TG are urgently required to better estimate the incidence in this population and to better prepare for the next generation of HIV interventions among them.

Interest in trail participation was quite high, except for circumcision studies, suggesting, as iPrEx demonstrated, that this is a suitable population for future generations of HIV preventive interventions. Northern Thailand may be a unique context in which to study HIV preventive interventions for Transgender populations.

The findings of high HIV prevalence and incidence among these populations of MSM in Chiang Mai are unfortunately consistent with data from other Thai sites, and from the wider Southeast and East Asian region.^1,2^ van Griensven, et al, reported even higher rates of HIV infection among MSM in Bangkok, and used robust sampling methods to identify very high rates of incident HIV among MSM at multiple venues. ^1^ A recent review of the global epidemiology of HIV among MSM found that while Thailand had the highest HIV rates among MSM, a number of other Asian countries also had serious epidemics: these included Vietnam with 6.2% prevalence (95% CI 5.1–7.3); Laos at 5.4% (95% CI 3.5–7.2); Cambodia with 7.8% (95% CI 5.9–9.7); Indonesia at 9.0% (95%CI 6.9–11.0); China at 4.3% (95% CI 4.0–4.7) and India at 14.5% (95% CI 13.3–15.6).^2^ In each of these countries, HIV rates among general population samples are below 1.0, except Thailand, at 1.18% of reproductive age adults. ^2^ Across Asia, then, MSM are at markedly increased risk for HIV infection compared to other men and women of reproductive age. The consistency of this finding is striking and underscores the urgent need for more effective HIV prevention tools for MSM in Asia. Thailand with its long history of support for collaborative HIV prevention research will likely play important roles in the development, testing, and implementation of these new strategies.
